# Assessment of Post-COVID-19 Changes in Brain—Clinical and Imaging Evaluation Using MRI Vessel Wall Imaging and Complementary MRI Methods

**DOI:** 10.3390/jcm13226884

**Published:** 2024-11-15

**Authors:** Jakub Okrzeja, Maciej Alimowski, Adam Garkowski, Marcin Hładuński, Bożena Kubas, Justyna Adamczuk, Piotr Czupryna, Karolina Narejko, Anna Moniuszko-Malinowska

**Affiliations:** 1 Department of Radiology, Medical University of Bialystok, 15-276 Białystok, Poland; adam.garkowski@gmail.com (A.G.); marcin.hladunski@umb.edu.pl (M.H.); bozenakubas06@gmail.com (B.K.); 2 Doctoral School of Social Sciences, University of Bialystok, 15-328 Białystok, Poland; m.alimowski@uwb.edu.pl; 3 Department of Infectious Diseases and Neuroinfections, Medical University of Bialystok, 15-540 Białystok, Poland; justyna.adamczuk@umb.edu.pl (J.A.); avalon-5@wp.pl (P.C.); knarejko1@umb.edu.pl (K.N.); annamoniuszko@op.pl (A.M.-M.)

**Keywords:** vessel wall imaging, VWI, magnetic resonance imaging, COVID-19, post-COVID-19 syndrome

## Abstract

**Background/Objectives**: The aim of the study was to evaluate the usefulness of vessel wall imaging and MRI in assessment of the post-COVID-19 changes in the brain. VWI is a progressive MRI technique that provides precise imaging of the pathological process in the wall of the vessel. It might help us to better understand the pathophysiology of COVID-19-related neurological lesions and may have an impact on management protocols. **Methods**: A total of 43 patients were included in the study; the post-COVID-19 group included 23 patients hospitalized for COVID-19 (mean age of the group: 53.52 years; 26% male, 74% female). The control group consisted of 20 patients from the general population who did not suffer from COVID-19 (mean age: 52.15 years; 35% male, 65% female). MRI examinations were performed on a 3T scanner (Biograph mMR, Siemens). The VW-MRI protocol included T1-weighted SPACE FS black-blood images, FLAIR images, SWI, and MRA. **Results**: Several radiological changes in post-COVID-19 patients were described: hyperintense foci in the white matter of the brain hemispheres, in the lower parts of the temporal lobes, and in the structures of the posterior cranial fossa; presence of engorgement of deep medullary veins or perivascular enhancement; presence of inflammatory vessel thickening in VWI images; changes in hippocampus size; presence of cortical atrophy; and thickening of the mucous membrane of the paranasal sinuses. The presence of atherosclerotic vessel thickening in VWI and the width of the third ventricle depended on the age of the patient. **Conclusions**: VWI and MRI may be useful in the assessment of post-COVID-19 lesions in the brain.

## 1. Introduction

The coronavirus disease 2019 (COVID-19) pandemic was caused by the severe acute respiratory syndrome coronavirus 2 (SARS-CoV-2) [1]. Additionally, the first cases of this illness were reported in Wuhan, Hubei Province, China [2]. SARS-CoV-2 is usually linked with pulmonary infection leading to pneumonia, but recent studies have shown that other organs might be affected, e.g., the nervous, gastrointestinal, cardiovascular, and immune systems [3,4].

COVID-19-related neurological disorders have been widely described around the world [4,5,6,7]. Among the many theories as to why these individuals present neurological symptoms and signs is an inflammatory or immunological process. Neuropathologic analyses have revealed that these patients have inflammatory mechanism affecting the endothelium [8,9].

It is also worth paying attention to the pathophysiology of brain involvement in COVID-19. The capability of SARS-CoV-2 to infect and replicate within the human brain has been conclusively proven through various techniques. These include the identification of genomic and subgenomic RNA via polymerase chain reaction (PCR), imaging methods that have visualized SARS-CoV-2 RNA and proteins within central nervous system (CNS) cells, and sequencing performed on CNS tissues [4]. Notably, research has demonstrated that SARS-CoV-2 can be present in many parts of the nervous system, such as both hemispheres, the brainstem, the thalamus, and the sciatic nerves, though it has not been detected in the dura mater [4]. SARS-CoV-2’s high affinity for the angiotensin-converting enzyme 2 receptor may allow it to directly infect the nervous system. This receptor is found in both neurons and glial cells, which could explain the neurological symptoms seen in some patients, such as loss of smell (anosmia), peripheral nerve damage, and disorders affecting brain function [4]. The most common pathologies of the cerebrum discovered in COVID-19 patients are dispersed ischemic/hypoxic damage, hemorrhagic and ischemic strokes, acute and subacute infarcts, encephalopathy, vascular congestion that may be connected to the hemodynamic changes caused by the infection, and dispersed and focal microglial activation with destruction of neurons by phagocytic cells mainly localized in the lower part of the brainstem [4]. Moreover, neuronal injury in the brainstem may result in a variety of clinical manifestations, such as abnormal cardiorespiratory regulation, lethargy, sleep abnormalities, and other clinical signs [4]. Therefore, it is probable that associations between the microglia and nerve cells are rather secondary to hypoxic/ischemic damage than to a direct reaction to SARS-CoV-2 infection of neurons [4].

When describing neurological problems in people with COVID-19, it is worth mentioning post-COVID-19 syndrome. Post-COVID-19 syndrome is defined by the World Health Organization (WHO) as “condition which occurs in individuals with a history of probable or confirmed SARS CoV-2 infection, usually 3 months from the onset of COVID-19 with symptoms and that last for at least 2 months and cannot be explained by an alternative diagnosis” [4]. The most common signs of neurological and neuropsychiatric post-COVID-19 syndrome are exhaustion, cognitive disorders such as brain fog, problems with memory, deterioration of concentration, anxiety, depression, and sleep disorders, which may be found in nearly one-third of patients 12 weeks following the beginning of acute COVID-19 disease [4]. Furthermore, these symptoms remain and are much more widespread in a short period (3–6 months) than in a long period of time (more than half a year after infection) [4].

When thinking about it, innovative imaging to properly examine the vessel wall becomes necessary, leading to the idea of vessel wall imaging (VWI). Unfortunately, there are only a few studies that demonstrate VWI findings in COVID-19 patients. Some studies have presented the detection of vasculitic lesions by VWI in infections of CNS, including viral ones [10,11,12]. The role of VWI in diagnosing vasculopathy in COVID-19, herpes encephalitis, HCV encephalopathy, and HIV encephalopathy has been reported in studies [13,14,15,16].

In our research, we aimed to assess the post-COVID-19 changes in the brain using innovative VWI and magnetic resonance imaging (MRI), which might help us to better understand the pathophysiology of COVID-19-related neurological lesions and may have an impact on the management protocol.

## 2. Materials and Methods

The study population consisted of 2 groups:

- Group I: post-COVID-19 group—23 patients who were hospitalized for COVID-19 after a positive SARS-CoV-2 real-time polymerase chain reaction (RT-PCR) or antigen test. They were assessed 6 months after the infection.
- Group II: control group—20 patients from the general population who did not suffer from COVID-19, with a negative SARS-CoV-2 RT-PCR or antigen test.

Patients from both groups had no previous history of neurological diseases, e.g., stroke, chronic headache, epilepsy, or head injury. The age range in group I was from 33 to 65 years, of which 17 people lived in the city and 6 people lived in the countryside. The age range in group II was from 36 to 65 years, of which 15 people lived in the city and 5 people lived in the countryside. Some of the patients in group I had comorbidities: 10 with hypertension; 4 with each of Hashimoto’s disease, hypothyroidism, osteoarthritis, and type II diabetes; 3 with each of varicose veins and kidney stones; 2 with each of asthma, hyperlipidemia, thyroid nodules, atrial fibrillation, and hepatic steatosis; and 1 with each of chronic obstructive pulmonary disease, anemia, gout, discopathy, nodular goiter, Lyme disease, and rheumatoid arthritis. The inclusion criteria for the study were: patients over 18 years of age, informed consent of the patient to participate in the study, and neurological symptoms after COVID-19.

### 2.1. VWI MRI Acquisition

The examinations were performed at the Independent Department, Laboratory of Molecular Imaging in 2022. MRI examinations were performed on a 3T scanner (Biograph mMR, Siemens, Erlangen, Germany) using a 16-channel mMR head/neck matrix coil. The vessel wall magnetic resonance imaging (VW-MRI) protocol included sagittal 3D T1-weighted SPACE FS black-blood images (TR 700 ms, TE 11 ms, voxels size of 0.5 × 0.5 × 0.5 mm, slice thickness 0.5 mm, FOV 235 mm) obtained before and after the administration of gadolinium contrast agent (0.1 mmol/kg, Gadovist, Bayer, Leverkusen, Germany), sagittal 3D T2 SPACE (TR 1400 ms, TE 149 ms, voxels size of 0.6 × 0.6 × 0.6 mm, slice thickness 0.6 mm, FOV 180 mm), sagittal 3D fluid-attenuated inversion recovery (FLAIR) images (TR 7500 ms, TE 369 ms, TI 2100, voxels size of 0.5 × 0.5 × 1 mm, slice thickness 1 mm, FOV 235 mm), axial susceptibility-weighted imaging (SWI) (TR 29 ms, TE 20 ms, voxels size of 0.2 × 0.2 × 1.5 mm, slice thickness 1.5 mm, FOV 220 mm), and 3D time-of-flight MR angiography (MRA) (TR 21 ms, TE 3.6 ms, voxels size of 0.3 × 0.3 × 0.5 mm, slice thickness 0.5 mm, FOV 200 mm).

### 2.2. Image Analysis

**Figure 1:**
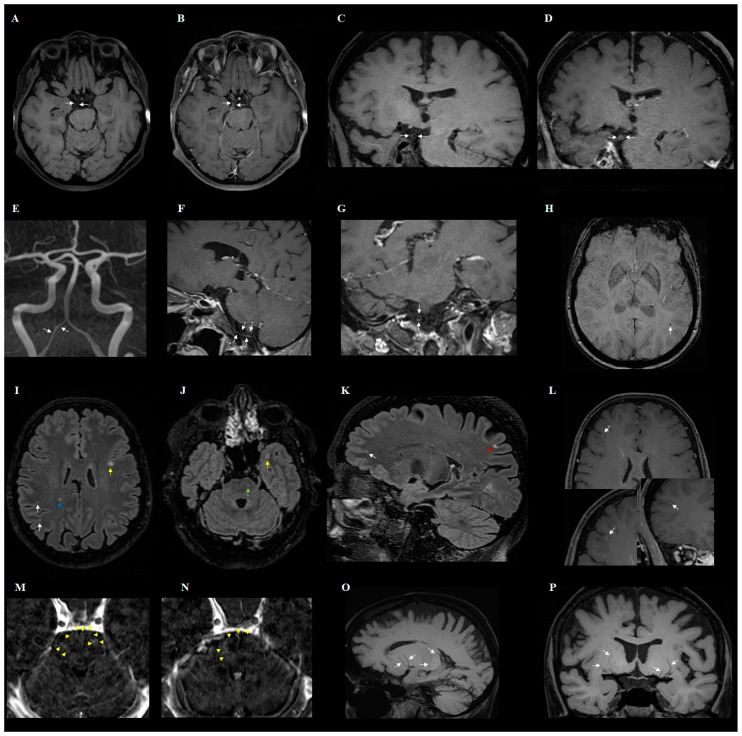
Magnetic resonance imaging of the brains of different patients with post-COVID-19 conditions. (**A**) Axial and (**C**) coronal sagittal pre-contrast 3D T1-weighted images, (**B**) contrast-enhanced axial, and (**D**) coronal sagittal 3D T1-weighted images show segmental concentric wall thickening and enhancement of the right fetal posterior cerebral artery (arrows), consistent with cerebral vasculopathy. (**E**) Coronal maximum intensity projection (MIP) reconstruction of the 3D TOF MR angiography demonstrates mild segmental narrowing of the right vertebral artery (arrows) with corresponding vascular contour irregularity, concentric thickening, and enhancement of vessel wall on the (**F**) sagittal and (**G**) coronal post-contrast 3D T1-weighted images (arrows). (**H**) SWI image shows a cerebral microhemorrhage in the subcortical white matter (arrow). (**I**,**J**) Axial and (**K**) sagittal 3D-FLAIR images demonstrate several hyperintensities with varying distributions—subcortical (white arrows), juxtacortical (yellow arrows), cortical (red arrow), and within the deep white matter (blue arrow) and pons (green arrow). (**L**) Axial and coronal post-contrast 3D T1-weighted images show small foci of cortical contrast enhancement in the right frontal lobe (arrows). (**M**,**N**) Axial contrast-enhanced 3D T1-weighted subtraction maps at the level of the pons demonstrate signal abnormalities along the course of perforating vessels (arrowheads). (**O**) Sagittal and (**P**) coronal minimum-intensity projection (MinIP) reconstruction of the pre-contrast 3D T1-weighted images at the level of the basal ganglia show bilateral tortuosity and mild dilatation of the perforator vessels (white arrows).

Radiologists assessed the following: hyperintense foci in the white matter of the brain hemispheres, in the paracortical area, in the cortex of the cerebral hemispheres, in the corpus callosum, in the lower parts of the temporal lobes (an unusual location for vascular changes caused by atherosclerosis), in the basal ganglia, in the thalamus, and in the structures of the posterior cranial fossa (brain stem, cerebellum); contrast enhancement in hyperintense foci; the presence of cerebral microbleeds in susceptibility-weighted imaging (SWI); the presence of engorgement of deep medullary veins; perivascular enhancement; enhancement of the pia mater in T1-weighted images (space black blood); the presence of inflammatory (concentric) and atherosclerotic (eccentric) vessel thickening in VWI images; hippocampus size according to the medial temporal lobe atrophy (MTA) scale; the presence of cortical atrophy (accentuation of pericerebral fluid spaces); the global cortical atrophy (GCA) scale; the width of third ventricle (in mm); and thickening of the mucous membrane of the paranasal sinuses [17] (Figure 1). Analysis was conducted independently by two experienced radiologists who were blinded from clinical details and other imaging findings.

### 2.3. Statistical Methods

Statistical analyses were performed using the R programming language [18]. All necessary data transformations were performed using the “tidyverse” package [19]. Statistical significance was determined using a significance level of α = 0.05, where a p-value below this threshold was considered important. The relationship between continuous variables was examined using Pearson’s correlation coefficient and point-biserial correlation for the relationships between continuous variables and dichotomous variables.

### 2.4. Ethical Issues

Ethical approval for this study was provided by the Ethics Committee on 16 December 2021 (APK.002.510.2021) and 24 October 2024 (APK.002.414.2024). The study was conducted in accordance with the Declaration of Helsinki, and all participants gave written informed consent. No compensation was provided for participation in this study, and the researchers were not compensated for their work.

## 3. Results

### 3.1. Baseline Characteristics

In Group I, the mean age was 53.52 years (standard deviation = 9.76). In total, 26% of individuals were male, and 74% were female. The mean age of the patients in Group II was 52.15 years (standard deviation = 8.37). In terms of sex distribution in control sample, 35% individuals were male and 65% individuals were female.

### 3.2. Descriptive Statistics of Group I

In the case of Group I (i.e., the research group), it is worth mentioning the examination/test results from the hospitalization period. Data for only 17 patients were available from the time of hospitalization. Nine patients had fever (53%), seven individuals reported dyspnea (41%), thirteen patients had cough (76%), two people had smell disturbances (12%), and one person had taste disturbances (6%). Furthermore, three patients were treated with Remdesivir (18%), one patient was treated with Tocilizumab (6%), eight were treated with Dexaven (dexamethasone) (47%), fifteen were treated with Heparin (prophylactically) (88%), and eight patients were treated with a nasal oxygen cannula (47%). The remaining results are shown in Table 1 and Table 2.

### 3.3. Descriptive Statistics of Group II

In Group II (control group), laboratory tests were not performed as in Group I because it was a group of healthy people (not hospitalized due to COVID-19). Compared to Group I, Group II showed higher mean results for data such as the presence of atherosclerotic (eccentric) vessel thickening in VWI images. Furthermore, compared to Group I, Group II demonstrated lower mean results in data such as hyperintense foci in the white matter of the brain hemispheres, hyperintense foci—paracortical or in the cortex of the cerebral hemispheres, hyperintense foci in the lower parts of the temporal lobes, hyperintense foci in the structures of the posterior cranial fossa (brain stem, cerebellum), presence of engorgement of deep medullary veins or perivascular enhancement, presence of inflammatory (concentric) vessel thickening in VWI images, hippocampus size according to the MTA scale, presence of cortical atrophy (accentuation of pericerebral fluid spaces), GCA scale, width of the third ventricle (in mm), and thickening of the mucous membrane of the paranasal sinuses [20]. These radiological findings are presented in Table 3.

### 3.4. The Pearson Correlation of Radiological Features with Various Clinical Data in Group I

During the hospitalization of patients from Group I, a significant amount of clinical and laboratory data were evaluated, which were used to create a correlation of radiological findings with these clinical data. The Pearson correlation was used for this statistical analysis. Statistically significant variables for the research sample are summarized in Table 4.

### 3.5. Point-Biserial Correlation of Radiological Features with Various Clinical Data in Group I

Moreover, we also made one more correlation of radiological features with clinical/laboratory data from the hospitalization of individuals from Group I. The point-biserial correlation was used for this statistical analysis. Statistically significant variables for the research sample are summarized in Table 5.

## 4. Discussion

Neurological disorders after COVID-19 represent one of the most important long-term worldwide public health issues involving both hospitalized and non-hospitalized people. An age above 65 is one of the most important risk agents for SARS-CoV-2 infection-related sequelae with neurological problems [4]. The mean age of the patients in the research sample who participated in our study, the aim of which was to assess post-COVID-19 changes, was 53.52 ± 9.76 years. Some cases in the literature of neurological disorders after COVID-19 in which imaging was used describe that patients with lesions in the CNS ranged from 11 to 88 years of age [4,21].

Post-COVID-19 smell and taste disorders have been frequently described. Studies indicate that, during the early stages of infection, around 60–70% of patients experience these symptoms. Specifically, one study found that 64.3% of COVID-19 patients reported changes in their sense of smell or taste, with this number gradually declining over time [22,23,24]. In our patients, only 9% reported an impaired sense of smell and taste. This result differs from the results found in some articles because our study included a small number of patients, as we wanted to focus more on radiological findings.

Early detection of brain lesions and the involvement of vessels in COVID-19 are significant, as it may lead to the early initiation of treatment with, e.g., anti-inflammatory and anti-platelet medications to prevent the appearance of complications. Therefore, imaging, such as VWI and MRI, plays a crucial role in detecting lesions in the CNS, as well as vascular involvement.

Nevertheless, typical imaging techniques such as MR angiography, computed tomography (CT) angiography, and digital subtraction angiography provide data about luminal involvement only, in the form of stenosis, beading, or irregularity [25]. VWI is an excellent technique for directly evaluating lesions of the walls of vessels by suppressing the cerebrospinal fluid (CSF) and luminal blood, which makes the vascular wall prominent and, hence, useful in diagnosing secondary viral vasculitis, e.g., in COVID-19 [25,26]. The wall thickness of intracranial vessels is between 0.2 and 0.3 mm. This thickness is still lower than the voxel size of presently accessible imaging software. However, the vascular wall may be visualized by appropriately suppressing CSF and blood within the voxel using different techniques [25,26].

A higher magnetic field (3 T over 1.5 T) is preferred for VWI due to the better signal-to-noise ratio. Therefore, we used the Biograph mMR 3T simultaneous scanner (Siemens) in our study. Imaging may be acquired in 2D mode or 3D mode. The benefits of 3D images include a high signal-to-noise ratio, isotropic voxels, multiplanar scans, greater suppression of signal of arteries, and a low specific absorption rate [25]. Isotropic voxels allow us to visualize lesions at many levels. However, the benefits of 2D scans include higher in-plane spatial resolution, sharper imaging due to decreased signal decay, and less susceptibility to motion artifacts [25]. Because of the anisotropic nature, partial volume impacts are a disadvantage of 2D imaging [25].

Moving on to correlations, the Pearson correlation of age turned out to be statistically significant. Zhuang FJ et al. reported that the severity of white matter hyperintensities increases with age [27]. This analysis indicates that aging is an independent contributor to the prevalence and severity of white matter hyperintensities [27]. Furthermore, the Pearson correlation of the width of the third ventricle (in mm) and age turned out to be statistically significant as well. Guenter W et al. described that age and the width of the third ventricle are strongly related and are associated with brain atrophy [28]. Furthermore, age and the width of the third ventricle are strong predictors of cognitive impairment (which may be connected with COVID-19 complications) [28].

Moreover, the point-biserial correlation of presence of atherosclerotic (eccentric) vessel thickening in VWI and age turned out to be statistically significant. Cogswell PM et al. reported that a small but statistically significant increase in vessel wall thickness and outer vessel wall diameter is found with age, which might represent early atheromatous lesions without discrete plaque formation [29]. These changes may be related to COVID-19. This statement is confirmed by research which has presented that VWI may detect inflammation and structural lesions in the walls of cerebral arteries, especially in cases of COVID-19-associated vasculopathy. Findings from VWI include vessel wall thickening and enhancement, which are thought to be associated with inflammatory processes in the vascular system caused by the virus. These lesions have been observed in several brain areas (e.g., basilar artery, middle cerebral artery, posterior cerebral artery), providing valuable insight into COVID-19’s impact on the CNS [30].

Interestingly, Mazzacane F et al. demonstrated that concentric vessel wall thickening in patients with COVID-19 in VWI, with associated stenosis and/or occlusion on magnetic resonance angiography and cerebral angiography, may be connected with cryptogenic stroke [31]. However, it is worth mentioning that, despite the VWI showing thickening of vessels and changes that may suggest inflammation in our patients, it is difficult to say at this point that performing this examination in patients with post-COVID-19 lesions in the brain makes more sense. In typical CNS vasculitis, changes in VWI are much more extensive than in post-COVID-19 syndrome [29,31].

Our study suffers from the limitation of a small study sample. Some of the results may be biased because correlations might have been influenced by multiple factors (e.g., age of the patients, few detected changes from which correlations cannot be made). Large-scale studies are warranted to prove the role of VWI in the imaging of post-COVID-19 lesions.

## 5. Conclusions

1. The changes observed in post-COVID-19 patients were: hyperintense foci (in the white matter of the brain hemispheres, in the lower parts of the temporal lobes, and in the structures of the posterior cranial fossa), presence of engorgement of deep medullary veins or perivascular enhancement, presence of inflammatory (concentric) vessel thickening in VWI images, changes in hippocampus size according to the MTA scale, presence of cortical atrophy, and thickening of the mucous membrane of the paranasal sinuses.
2. In addition to the characteristic changes in COVID-19, it was observed that the presence of atherosclerotic (eccentric) vessel thickening in VWI and the width of the third ventricle depend on the age of the patient.
3. Despite thickening of vessels and changes that might suggest inflammation in VWI in post-COVID-19 patients, lesions in typical CNS vasculitis are much more extensive than in post-COVID-19 syndrome. Large-scale studies are warranted to demonstrate the role of VWI in imaging of post-COVID-19 changes.

## Figures and Tables

**Figure 2:**
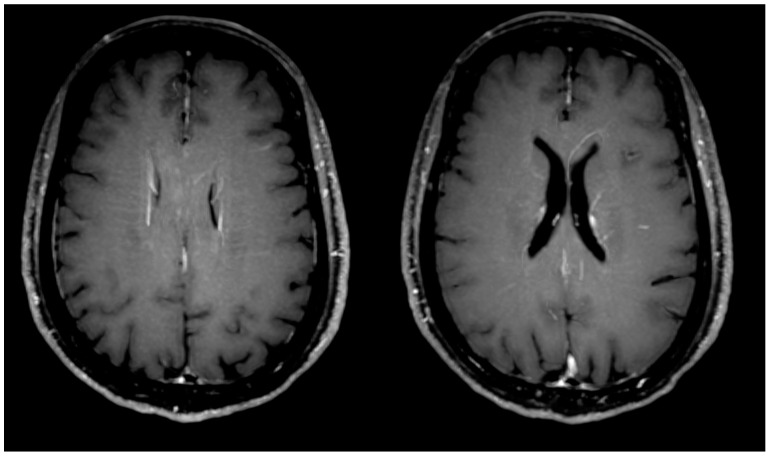
Axial MIP reconstruction of the post-contrast 3D T1-weighted images at the level of lateral ventricles shows bilateral perivascular enhancement along engorged deep medullary veins.

**Table 1:** Group I descriptive statistics: radiological results.

Radiological Results	Mean	SD	SE	Range
Hippocampus size according to the MTA scale	0.83	0.58	0.12	(0–4)
GCA scale	0.83	0.39	0.08	(0–3)
Width of third ventricle (in mm)	4.93	1.88	0.39	(2–9)
**Radiological Results**	**n**		**%**	
Hyperintense foci in the white matter of the brain hemispheres	20		87%	
Hyperintense foci—paracortical or in the cortex of the cerebral hemispheres	6		26%	
Hyperintense foci in the corpus callosum	3		13%	
Hyperintense foci in the lower parts of the temporal lobes	7		30%	
Hyperintense foci in the basal ganglia or in the thalamus	3		13%	
Hyperintense foci in the structures of the posterior cranial fossa (brain stem and cerebellum)	7		30%	
Presence of cerebral microbleeds in SWI	3		13%	
Presence of engorgement of deep medullary veins or perivascular enhancement (Figure 2)	3		13%	
Enhancement of the pia mater in T1-weighted images (space black blood)	3		13%	
Presence of inflammatory (concentric) vessel thickening in VWI images	7		30%	
Presence of atherosclerotic (eccentric) vessel thickening in VWI images	11		48%	
Presence of cortical atrophy (accentuation of pericerebral fluid spaces)	19		83%	
Thickening of the mucous membrane of the paranasal sinuses	15		65%	

**Table 2:** Group I descriptive statistics: laboratory/clinical results.

Laboratory/Clinical Results	Mean	SD	SE	Range
SpO_2_—beginning of hospitalization	90.62	5.98	1.49	(78–96)
Remdesivir—duration of treatment	0.71	1.57	0.38	(0–4)
Tocilizumab—duration of treatment	0.06	0.24	0.06	(0–1)
Dexaven—duration of treatment	4.59	6.1	1.48	(0–20)
Heparin (prophylactically)—duration of treatment	7.18	4.25	1.03	(0–16)
WBC—beginning of hospitalization (in count per microliter)	5.83	1.97	0.48	(2.74–8.84)
Neutrophils—beginning of hospitalization (in count per microliter)	3.88	2.02	0.49	(1.31–8.17)
Lymphocytes—beginning of hospitalization (in count per microliter)	1.4	0.77	0.19	(0.36–3.26)
PLT—beginning of hospitalization (in count per microliter)	211.41	62.95	15.27	(125–379)
AST—beginning of hospitalization (in IU/L)	40.24	36.95	8.96	(15–154)
ALT—beginning of hospitalization (in IU/L)	45.06	43.17	10.47	(10–168)
Creatinine—beginning of hospitalization (in mg/dL)	0.75	0.22	0.05	(0.48–1.35)
D-dimers—beginning of hospitalization (in µg/L)	755.4	521.67	134.69	(160–1811)
Ferritin—beginning of hospitalization (in µg/L)	695.17	853.43	220.35	(10.9–2838)
Fibrinogen—beginning of hospitalization (in mg/dL)	483.47	153.8	37.3	(242–824)
WBC—end of hospitalization (in count per microliter)	6.73	1.98	0.51	(3.77–10.73)
Neutrophils—end of hospitalization (in count per microliter)	3.75	1.57	0.4	(1.65–6.57)
Lymphocytes—end of hospitalization (in count per microliter)	2.11	0.68	0.18	(1.31–3.37)
PLT—end of hospitalization (in count per microliter)	255.53	99.33	25.65	(134–429)
AST—end of hospitalization (in IU/L)	32.31	15.64	4.34	(17–65)
ALT—end of hospitalization (in IU/L)	46.17	21.57	6.23	(15–87)
Creatinine—end of hospitalization (in mg/dL)	0.74	0.14	0.04	(0.53–0.98)
D-dimers—end of hospitalization (in µg/L)	632	477.46	123.28	(157–2005)
Ferritin—end of hospitalization (in µg/L)	782.03	762.25	254.08	(9.6–2556)
Fibrinogen—end of hospitalization (in mg/dL)	349.75	89.78	25.92	(214–501)
Length of hospitalization (days)	8.53	4.6	1.12	(1–20)
How many doses of the vaccine?	1	1.12	0.25	(0–3)
**Laboratory/clinical Results**	**n**		**%**	
Post-COVID-19 headaches	6		26%	
Post-COVID-19 vertigo	7		30%	
Post-COVID-19 memory disorders	16		70%	
Post-COVID-19 concentration disorders	6		26%	
Post-COVID-19 dyspnea	10		43%	
Post-COVID-19 cough	2		9%	
Post-COVID-19 smell disorders	2		9%	
Post-COVID-19 taste disorders	2		9%	
Post-COVID-19 thromboembolic episode	9		39%	
Vaccinated against COVID-19	10		43%	

**Table 3:** Group II descriptive statistics.

Radiological Results	Mean	SD	SE	Range
Hippocampus size according to the MTA scale	0.4	0.5	0.11	(0–1)
GCA scale	0.75	0.44	0.1	(0–1)
Width of third ventricle (in mm)	4.28	1.85	0.41	(2–8)
**Radiological Results**	**n**		**%**	
Hyperintense foci in the white matter of the brain hemispheres	15		75%	
Hyperintense foci—paracortical or in the cortex of the cerebral hemispheres	2		10%	
Hyperintense foci in the lower parts of the temporal lobes	4		20%	
Hyperintense foci in the structures of the posterior cranial fossa (brain stem and cerebellum)	3		15%	
Presence of engorgement of deep medullary veins or perivascular enhancement	1		5%	
Presence of inflammatory (concentric) vessel thickening in VWI images	1		5%	
Presence of atherosclerotic (eccentric) vessel thickening in VWI images	11		55%	
Presence of cortical atrophy (accentuation of pericerebral fluid spaces)	15		75%	
Thickening of the mucous membrane of the paranasal sinuses	10		50%	

**Table 4:** Correlation of radiological findings of Group I with various clinical/laboratory data (the Pearson correlation).

Radiological Features	Clinical Data	Pearson Correlation Coefficient	*p* Value
Width of third ventricle (in mm)	Age	0.62	0.01
Width of third ventricle (in mm)	Dexaven—duration of treatment	0.56	0.02
Width of third ventricle (in mm)	Dexaven	0.56	0.02
Width of third ventricle (in mm)	Nasal oxygen cannula	0.52	0.03

**Table 5:** Correlation of radiological findings of Group I with various clinical/laboratory data (the point-biserial correlation).

Radiological Features	Clinical Data	Correlation Coefficient	*p* Value
Hyperintense foci in the structures of the posterior cranial fossa (brain stem, cerebellum)	Dexaven—duration of treatment	0.76	0.01
Hyperintense foci in the structures of the posterior cranial fossa (brain stem, cerebellum)	Heparin (prophylactically)—duration of treatment	0.72	0.01
Presence of atherosclerotic (eccentric) vessel thickening in VWI images	Age	0.59	0.01
Hyperintense foci in the structures of the posterior cranial fossa (brain stem, cerebellum)	Dexaven	0.59	0.01
Hyperintense foci in the structures of the posterior cranial fossa (brain stem, cerebellum)	Oxygen nasal cannula	0.59	0.01
Hyperintense foci in the corpus callosum	Heparin (prophylactically)—duration of treatment	0.56	0.02
Hyperintense foci in the structures of the posterior cranial fossa (brain stem, cerebellum)	SpO_2_—beginning of hospitalization	−0.56	0.02
Presence of inflammatory (concentric) vessel thickening in VWI images	Tocilizumab	0.54	0.03
Presence of inflammatory (concentric) vessel thickening in VWI images	Tocilizumab—duration of treatment	0.54	0.03
Hyperintense foci in the lower parts of the temporal lobes	Dexaven	0.54	0.03
Hyperintense foci in the lower parts of the temporal lobes	Dexaven—duration of treatment	0.53	0.03
Hyperintense foci in the corpus callosum	Dexaven—duration of treatment	0.52	0.03
Hyperintense foci in the lower parts of the temporal lobes	SpO_2_—beginning of hospitalization	−0.51	0.04
Presence of inflammatory (concentric) vessel thickening in VWI images	Oxygen nasal cannula	0.49	0.05

## Data Availability

Data will be made available on request.
